# Ultrasound-guided Mid-humeral Radial Nerve Block Provides Sufficient Surgical Anesthesia at Hand Dorsum: A Novel Method and Report of Three Cases

**DOI:** 10.7759/cureus.3949

**Published:** 2019-01-24

**Authors:** Onur Balaban, Tayfun Aydın, Sermet İnal, Merve Yaman

**Affiliations:** 1 Anesthesiology, Kutahya Health Sciences University, Kutahya, TUR; 2 Orthopaedics, Kutahya Health Science University, Kutahya, TUR

**Keywords:** ganglion cyst, mid-humeral radial nerve block, ultrasound

## Abstract

A peripheral nerve block is a sufficient anesthesia method for hand surgeries, as it protects the patient from the complications of general anesthesia and provides postoperative analgesia. We report the first use of an ultrasound-guided mid-humeral radial nerve block in three cases of ganglion cyst excision from hand dorsum. The block provided efficient surgical anesthesia and postoperative analgesia for the excision of ganglion cysts at the hand dorsum.

## Introduction

Ganglion cysts are soft tissue masses that arise from the joint capsule and tendons which are commonly seen on the dorsal side of the hand and wrist [1]. One of the treatment options is surgical excision [2]. Surgery is usually performed with local anesthesia infiltration in sedated-unsedated patients or under general anesthesia.

In recent years, ultrasound (US) guidance has made distal peripheral nerve blocks of the upper extremity technically safe and feasible options to achieve anesthesia for hand and wrist surgery [3]. Nowadays, US-guided regional blocks should be the first choice for such surgical procedures where the possible risks of general anesthesia and the necessity of clear airways are considered [4]. US-guided nerve blocks have many advantages, such as the avoidance of nerve damage, with a clear definition of nerves from the surrounding structures, control of the distribution of local anesthetics, visualization of needle position, faster block onset time, improved block qualities, and a reduction in the volumes of local anesthetics [5-6]. The success rate of regional anesthesia has increased when performed under ultrasound guidance. Ultrasound identifies nerves easily, which cannot be defined by surface anatomic landmarks and alternative sites can be determined by scanning the nerve along its route [6-7].

Forearm blocks may especially be useful for minor surgeries of the hand. However, background data are limited regarding the performance of a peripheral nerve blockade at the level of the upper arm for hand surgery. There is also a lack of reports about the optimal sites for needle insertion regarding US-guided radial nerve blocks. Most studies about median, ulnar, and radial nerves present these blocks as rescue techniques for a failed or incomplete proximal (infraclavicular, axillary, interscalene, or supraclavicular) upper extremity block [4,8-11].

The mid-humeral region enables anesthesiologists to selectively administer local anesthetics to different nerves and block the four main nerves of the upper extremity separately [12]. This technique may have advantages over proximally performed approaches, such as the avoidance of needle trauma to central structures, decreased motor blockade, and smaller amounts of local anesthetic drugs used to achieve anesthesia for a narrower area [4].

In this case report, we present three cases of US-guided radial nerve blocks performed at the mid-humeral region. Efficient surgical anesthesia was achieved for ganglion cyst excision at the hand dorsum using the minimal dose of local anesthesia. We discuss the potential indications and advantages of a US-guided radial nerve block at the mid-humeral region for patients undergoing hand surgery.

## Case presentation

**Figure 1:**
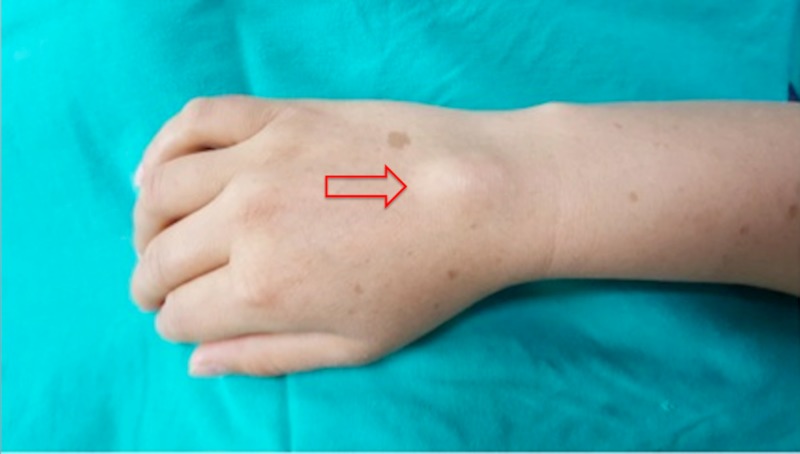
A ganglion cyst at the hand dorsum Arrow indicates the 2x3-centimeter-sized mass

Written informed consent has been obtained from all patients for the anesthetic/surgical procedures and for the publication of this case report. All patients underwent the excision of ganglion cysts at the dorsum of the hand (Figure 1).

**Figure 2:**
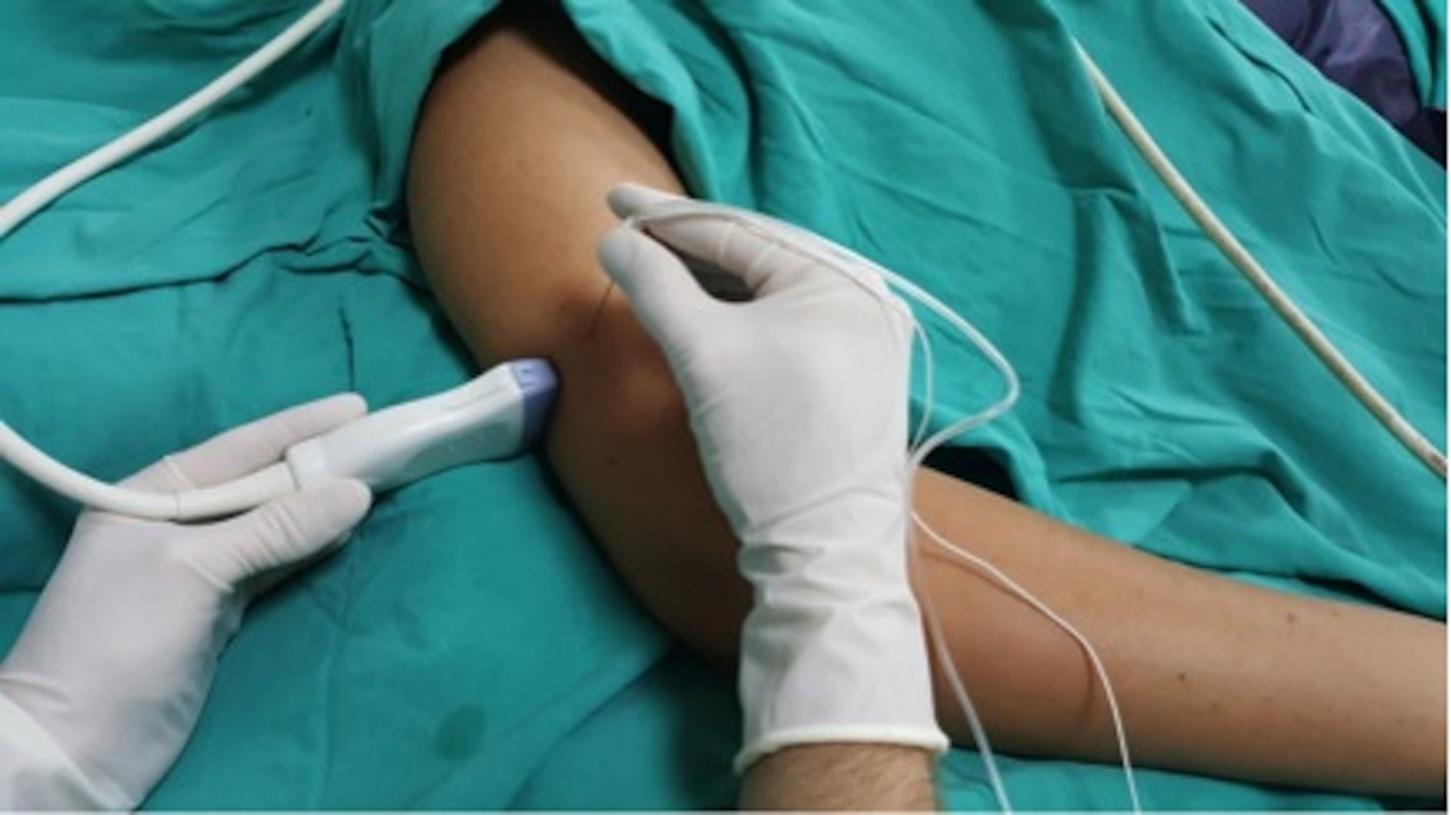
Scanning position of the ultrasound probe and position of the needle during the mid-humeral radial nerve block

Standard monitorization (electrocardiogram, blood pressure, pulse oximetry) was applied at the block room. An intravenous cannula was placed and infusion of 0.9% NaCl solution was started. The patients were sedated by administering 0.03 mg/kg of midazolam and 0.5 µg/kg of fentanyl intravenously. After sedation, the position of the arm was set at the 90 degrees abduction of the arm and 90 degrees of the forearm on the surgery table. The ultrasound probe was placed transversally at the mid-humeral region, on the posterolateral aspect of the arm. The needle was entered from the lateral side of the arm in the medial direction within the plane of the ultrasound beam (Figure 2).

**Figure 3:**
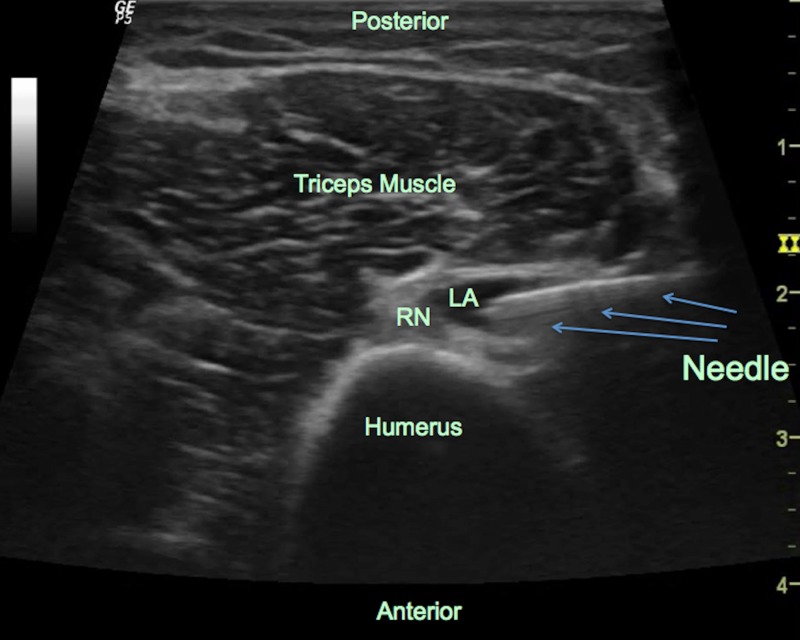
The ultrasonographic view of the radial nerve, the block needle, and local anesthetic spread around the nerve RN: radial nerve, LA: local anesthetic; arrows indicate the needle position during local anesthetic injection

The block area was sterilized with iodine solution and the US transducer was covered with a sterile cap. We performed needle insertion at the mid-humeral region, which is located midway between the anterior process of the acromion and the lateral epicondyle of the humerus, as described by Foxall et al. [6]. The radial nerve could be visualized easily by locating the ultrasound probe on the posterolateral aspect of the arm at this level. A 13 MHz linear transducer was used (LOGIQ P5®, General Electric, USA). The radial nerve was visualized as an oval, heterogeneous structure that consisted of hypoechoic and hyperechoic structures, which represent the nerve fascicles and connective tissue. Using the in-plane technique, a 22 Ga 8 cm echogenic needle (Stimuplex® Ultra 360® Braun, Melsungen, Germany) was introduced into the mid-humeral region, aiming to enter the fascial plane next to the radial nerve. After the localization of the needle tip on the radial nerve, 0.15 ml/kg of 0.5% bupivacaine was injected. The distribution of the local anesthetic drug and the tip of the needle was visualized in real time during the procedure, aiming to spread around the nerve (Figure 3).

**Figure 4:**
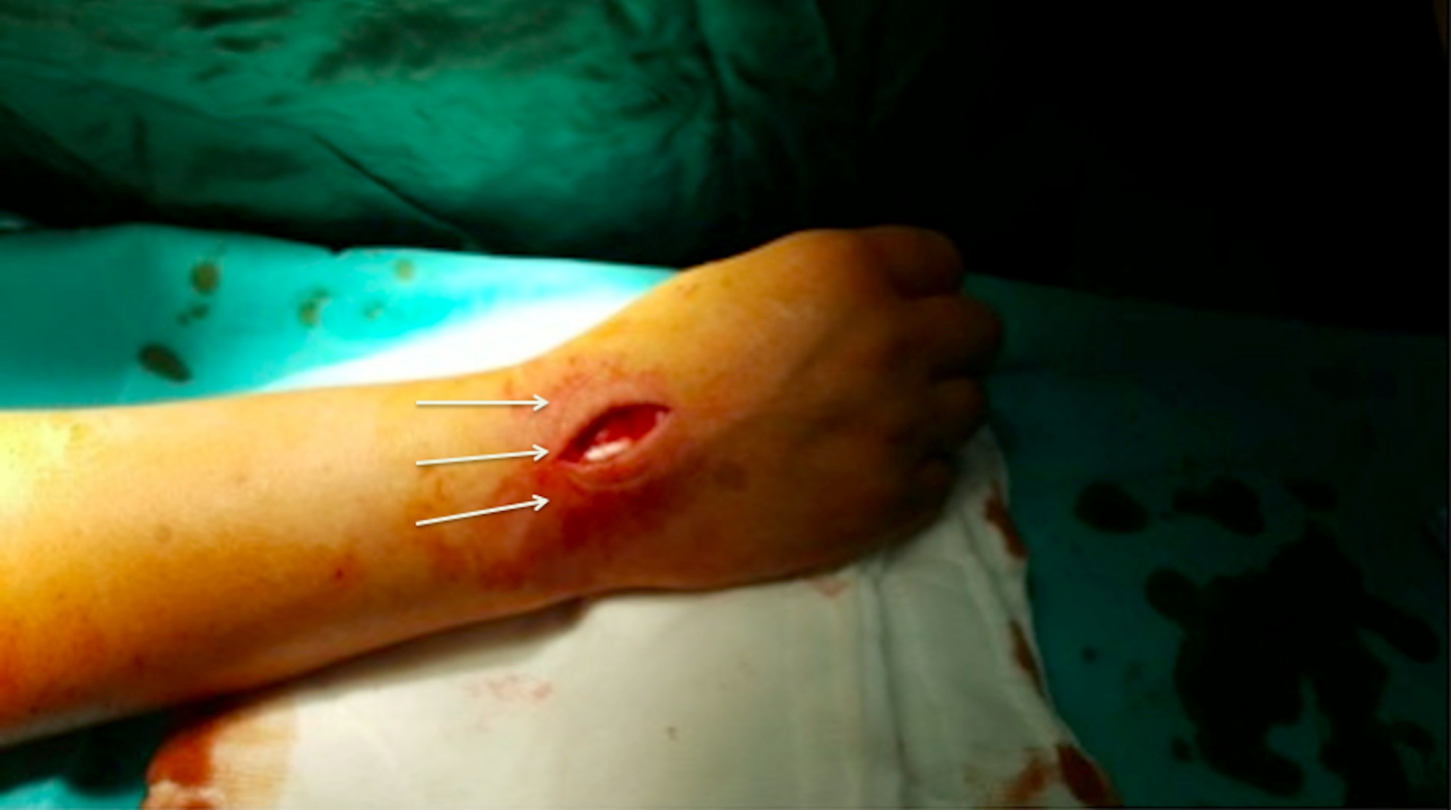
The incision performed for excision of the ganglion cyst

We did not use a nerve stimulator, as the nerve was clearly identified under ultrasound guidance. The patients were followed up with pinprick tests after the blocks for loss of sensation. The incisions were within the sensory dermatomal innervation area of the radial nerve (Figure 4).

Case 1

A 33-year-old female patient presented with complaints of swelling and pain in the dorsum of the right hand. She had a 2x2 cm mass and a soft cystic lesion, which was diagnosed as a ganglion cyst at the dorsum of the hand (Figure 1). Past medical history was unremarkable with American Society Anesthesiology (ASA) classification I. Surgical history examination revealed two cesarian-section operations. Ten milliliters of local anesthetic (bupivacaine 0.5%) was administered to encircle the radial nerve without entering the humeral side. Surgical anesthesia was achieved at the 25th minute after local anesthetic administration. A mild motor block was observed, as the patient could move her hand parallel to gravity. The patient was cooperative and reported minor discomfort during the excision of the cyst from its base where it originated, but there was no need for additional analgesic drugs during the surgery. There were no symptoms of cardiovascular, respiratory, or central nervous system side-effects. The surgery proceeded uneventfully and lasted about 20 minutes. The block was considered successful without the necessitation of conversion to general anesthesia.

Case 2

A 28-year-old female patient with complaints of swelling in the wrist dorsum of the right hand. The patient's medical and surgical history was unremarkable and evaluated as ASA I class. Fifteen milliliters of local anesthesia (10 ml bupivacaine 0.5 % and 5 ml lidocaine 2%) was administered around the radial nerve under ultrasound guidance. The block procedure was uneventful. The patient was cooperative during the operation and did not report pain at the beginning of the surgery. During the excision of the cyst from its base, the patient complained of discomfort. Fentanyl 50 µg intravenous was administered and 3 milliliters of 2% prilocaine was infiltrated to the surgical area. The surgery lasted 30 minutes, uneventfully. The block was considered successful without the need for conversion to general anesthesia.

Case 3

A 24-year-old male patient with a complaint of swelling at the wrist dorsum of the right hand was diagnosed with a ganglion cyst. The patient was evaluated as ASA I class with no remarkable medical and surgical history. After identifying the radial nerve under ultrasound guidance, 10 milliliters of 0.5% bupivacaine was administered. There were no symptoms of side effects during the block procedure. The patient reported minor discomfort, which was resolved with the administration of 50 µg intravenous fentanyl and infiltration of 3 milliliters 2% prilocaine into the surgical area. The surgical procedure was completed in 30 minutes without any complications. The block was considered successful, with no need of conversion to general anesthesia.

## Discussion

In this case report, a US-guided radial nerve block was applied from the mid-humeral level, which allowed sufficient surgical anesthesia for the excision of the ganglion cyst from the dorsal side of the hand. The course of the radial nerve in the distal part of the upper arm has a great variety [13]. The radial nerve passes obliquely across the back of the humerus between the lateral and medial heads of the triceps. It enters the anterior compartment from the deltoid tuberosity to the lateral epicondyle and passes between the brachioradialis and brachialis muscles [6]. It usually divides approximately 2-3 cm proximal to the elbow. The radial nerve can be easily visualized as a single structure 5 cm proximal to the elbow lateral to the humerus and curves posterior at about 15 cm proximal from the medial epicondyle [7,13]. The nerve could be scanned at this level and can be found slightly posterolateral. The deep branch is sometimes difficult to visualize by ultrasound [14]. The superficial radial nerve is cutaneous, whereas the deep branch forms the posterior interosseous nerve and mainly supplies motor function. The deep branch also supplies sensation to the interosseous membrane, the periosteum of the radius and ulna, and the extensor surfaces of the carpal joints [6].

At the mid-humeral level, the four main nerves are anatomically well-separated. This allows for the selective administration of local anesthetics on both the radial and the musculocutaneous nerves with the ulnar and median nerves [12]. Before its division into two terminal branches, a mid-humeral radial nerve block would produce more widespread anesthesia of the wrist and can be used for anesthesia in a wider variety of surgery [6]. Foxall et al. reported the ease of identification of the radial nerve at the distal third level of the arm [6]. We could easily visualize the radial nerve at the mid-humeral level just posterior to the humerus as a single, separate, and bright hyperechoic, oval-shaped structure. When it is slightly scanned to distally closer to the elbow, it becomes more distant to the humerus and can be blocked at this level, before it travels into the intermuscular septum.

Reports about ultrasound-guided radial nerve blocks in the literature are rare and limited to very few anecdotal reports. These reports are limited to performance in emergency departments to provide analgesia for the reduction of distal radius fractures [15-18]. Blocking the radial nerve at the mid-humeral region has the advantage of blocking the motor function that the radial nerve innervates as well, which may be preferable in many operations. However, in certain operations, such as tendon repair, preservation of motor function may be desired intraoperatively. The maintenance of motor function is an important advantage of distal nerve blocks over proximal blocks. A more distal nerve block may be preferred to block the sensory function of the nerve. Also, a differential infraclavicular block (by using a low-dose local anesthetic) may be performed in such cases.

The general advantages of distal peripheral nerve blocks could be expressed as: Distal approaches to upper extremity blocks need a lower amount of local anesthetic drugs with a blockade of a specific area. These approaches also avoid the risk of inadvertent puncture to critical structures, such as pleura or big vessels, which may occur when a proximal block is applied [3,5]. Distal peripheral nerve blocks allow the preservation of proximal muscle function of the upper limb and patients may be discharged earlier from the hospital [19]. These blocks may be suitable in patients where local anesthetic infiltration is contraindicated, such as in cases of infection or suspected malignancy. The surgical site anatomy is preserved and may provide for better operating conditions by not causing tissue distortion with infiltrated local anesthetic [4]. Selective blockade of the specific nerve may shorten the time for anesthetic administration [4]. The disadvantages are that distal nerve blocks would not prevent tourniquet pain. Peripheral nerve blocks may not always be sufficient for all surgical procedures on the forearm that involve wider surgical areas [3]. Among our cases, two cases needed additional local anesthetic infiltration during the surgery. This may be due to a wider surgical dissection that exceeded beyond the analgesic area of radial nerve block. A proximal, brachial plexus block is an alternative for operations of the hand in which larger surgical areas involve multiple peripheral nerve sensory dermatomes.

## Conclusions

Our experience with an ultrasound-guided mid-humeral radial nerve blockade suggests that this block provides successful surgical anesthesia and postoperative analgesia for the excision of dorsal-side ganglion cysts of the hand when applied from the mid-humeral region. This regional anesthesia technique may be a feasible and more comfortable alternative to general anesthesia for patients undergoing hand surgery. A mid-humeral radial nerve block has the advantage of requiring a lower amount of local anesthetic drug and less motor block of the arm, which is favorable for outpatient settings.
